# The Corpus Sanum

**Published:** 1894-02-17

**Authors:** 


### Studies in Applied Sociology.

### III.—THE CORPUS SANUM.

The proverb *mens sana in corpore sano* has become one of our stock of commonplaces, but how few have yet discovered the proportion which *mens* should bear to *corpus* to make the perfect man. It is generally conceded, however, that gymnastics have a distinct educational value. The Germans, most thoughtful of nations, have noted this, and gymnastics, or "turnen," as they call them, have a fixed place in their educational system. Every German schoolboy receives weekly lessons, and the school exhibitions of gymnastics are neater and more finished than any English non-professional performers could show. Yet the Germans are not a nation of athletes. Why is this? Because even in training the body you cannot eliminate the soul. The spirit of athletics, the joyous impulse, cannot be given in two lessons a week. They are only lessons after all, and our Teuton so regards them, and gives up his trapeze performances and his Latin grammar with equal goodwill. "The German boy," says an acute American observer, "has practically no games. At eighteen he knows a great deal more Latin and Greek, he can go through methodical exercises with his class on the bars or with the dumb-bells, probably a great deal better than his Anglo-Saxon brother, but he will throw a base-ball as awkwardly as a girl, and he would be homeless on the football field. A cricket ball he would mistake for a cannon ball, and consider it quite as deadly. The inevitable result of such a condition of things is that the young men, or rather boys, spend much time in quieter amusements, such as card-playing and billiards, and very often drink much beer as a preparation for their University course." On the whole, we think cricket and football, with all their faults, a better training for the work of life.

Yet some systematised form of gymnastics is useful. Growing boys overstrain themselves, delicate boys kill themselves, while striving to develop their muscles and improve their health. We are only beginning to realise this, and have had to borrow from Sweden a method of gymnastics duly adapted to every age and sex. Sweden is the home of scientific physical training. The founder of it was Peter Hendrick Ling, a native of the province of Smaland, and as the "Swedish" or "Ling" gymnastics the system is indifferently known. His invention was due to an accident. Suffering from an attack of gout in the arm he thought that exercise might cure it, and therefore began to fence. The idea was successful, and Ling then began to reflect on the possibilities of curing similar diseases by movement of the affected limbs. This also proved satisfactory, and then there dawned upon him the notion of the harmonious development of the body by means of exercises. In order to apply his theories accurately he studied anatomy and physiology, and in 1813, so famous and successful had he become, the Royal Central Institute of Gymnastics was established at Stockholm for the purpose of enabling him to carry out his ideas to the full. He was its director until his death in 1839.

The great difference between the Swedish and other systems of gymnastics is that muscular training is not the only nor the chief thing aimed at. The Swedish gymnast does not double up his arm to show you an abnormal biceps, but every part of the body, within and without, has its own exercise for the symmetrical development of the whole. The exercises are thus classified:—

1. Introductory.
2. Arch flexions, or movements of back and chest.
3. Heaving movements—lifting the body by the arms.
4. Balance movements, to give general equilibrium and a good carriage.
5. Shoulder-blade movements, to flatten the back.
6. Abdominal exercises, to aid digestion.
7. Lateral trunk movements.
8. Slow leg movements, to increase the circulation of the lower limbs and quiet the heart.
9. Jumping and vaulting.
10. Respiratory movements, to increase the capacity of the lungs.

Of these exercises it is probable that only No. 7 would find a place in any ordinary English notion of gymnastics. "Let the boy jump, run, swing, and climb, and let his digestion, lungs, and heart look after themselves. They will probably improve in the process." That is how most people regard the matter when they give it a thought at all. But these organs do not improve. "Show me one who has been an athlete, and who is more than 42 years' old," said a doctor recently, "and I will show you a prematurely old man." That this is too often the result of the Spartan training, the spurts of super-human exertion to which we give the name of athletics can hardly be denied by those who have watched the after career of athletes; but such a murderous development was not what was meant by gymnastics in ancient Greece, nor is it what results from the Swedish gymnastics to-day.

In almost all our public schools there is a gymnasium, but who is the teacher of gymnastics? Too often some old sergeant with military drill at his finger-tips—and certainly military drill has its value in teaching prompt obedience and harmonious action—but ignorant of gymnastic science. This is not enough. If we want our boys and girls to grow up strong and healthy men and women the various organs of the body must be scientifically and symmetrically developed, and this can be done only by teachers who know its form and functions, and not merely some tricks of agility belonging more to the circus than the school-room.

America is ahead of Britain in this matter. Mrs. Mary Hemenway, of Boston, in 1888 founded the Normal School of Gymnastics there, and her son, Mr. Augustus Hemenway, presented a gymnasium to Harvard University. Mrs. Hemenway began by teaching twenty-five women teachers. The next year she offered to bear the expense of training a hundred public school teachers, on condition that they introduced the Ling system into their school. This offer the School Board of Boston gratefully accepted. Later began the education of special teachers of physical training, and so popular has the system become that the demand for graduates and pupils of the school is greater than the supply. Failing a millionaire to provide, like Mrs. Hemenway, a training-school for gymnastics, is it too much to ask the Education Department to make some provision for the proper physical training of the children in the schools under its control?

